# Between experience and measurement: masters athletes’ perceptions of the 5Cs and a proposed measure

**DOI:** 10.3389/fspor.2026.1845368

**Published:** 2026-08-11

**Authors:** Maynara Priscila Pereira da Silva, Evandro Morais Peixoto

**Affiliations:** Graduate Program in Psychology, Sao Francisco University, Campinas, Brazil

**Keywords:** masters sport, positive psychology, psychological assessment, sport psychology, test construction

## Abstract

**Introduction:**

This study aimed to evaluate Masters athletes’ perceptions of the Cs of Positive Youth Development (competence, confidence, connection, care, character, contribution, and risk behavior) and to develop a measure to assess these dimensions in the context of Masters sport.

**Methods:**

The first stage included 25 Masters athletes from different sports, aged between 30 and 74 years, who completed a semi-structured interview and a sociodemographic questionnaire. Data were analyzed using the *Requalify*. *AI* platform to identify the main categories emerging from the interviews and to conduct a similarity network analysis. In the second stage, items corresponding to the emergent categories were developed and submitted to expert judges for content validation, yielding high content validity coefficients (CVC ≥ 0.90) for most items.

**Results:**

Initial findings suggest that masters sport is perceived as a context for social, emotional, physical, cognitive, and relational development, that is, articulating with Cs. However, some conceptual differences were observed between adolescents/young adults and Masters athletes, particularly regarding the notions of care and risk behavior. The results demonstrate theoretical and semantic coherence between the qualitative perceptions and the constructed items, providing evidence for the applicability of the 5Cs model to adult life.

**Discussion:**

The findings suggest that masters sport constitutes a favorable context for positive development, with the 5Cs remaining active and interdependent.

## Introduction

Over the past decades, the participation of adults and older individuals in competitive sports has increased substantially, promoting physical, emotional, and cognitive benefits (1–3). This movement, referred to as *masters sport*, has been associated with the enhancement of physical skills, improvements in health and physical fitness, stress relief, character development, and social integration, factors that contribute to active and healthy aging (4, 5). In this sense, masters sport extends beyond physiological or leisure-related benefits, emerging as a context for personal, relational, and moral development in adulthood (6, 7).

Masters sport is characterized by organized competitions involving athletes who initiate or resume their engagement in adulthood, typically from the age of 35 onwards, although the minimum age may vary depending on the sport, as observed, for example, in swimming (8). Notably, there is no upper age limit for participation, which reinforces its emphasis on athletic longevity (9).

The term “masters athlete” also encompasses the concepts of “senior athlete” and “veteran athlete,” as it includes individuals from diverse age groups who engage in sport activities governed by formal rules and varying levels of competition (9, 10). This practice is associated with sustained sport engagement across the lifespan and with the recognition of athletes’ accumulated experience (6). The expansion of national and international competitions specifically designed for this population, such as the World Masters Games, highlights the growth of a sociocultural movement that recognizes sport as a context capable of fostering personal, social, and moral fulfillment in adulthood (3, 11).

Although research on masters sport has gained increasing attention, it remains largely concentrated on qualitative studies aimed at understanding the subjective and social experiences of these athletes. Findings indicate that sport participation in adulthood is associated with psychosocial benefits, such as self-confidence, sense of belonging, emotional regulation, interpersonal connection, and autonomous motivation (10). These results suggest that sport may continue to function as a context for positive development in adulthood, extending what has been widely established in the field of Positive Youth Development (PYD) (12, 13).

The 5Cs model of Positive Youth Development (PYD), comprising competence, confidence, connection, character, and caring, has been widely used to understand the role of sport in the holistic development of adolescents and young athletes (13–16). However, emerging evidence suggests that these same dimensions are also manifested in adulthood, albeit with distinct meanings (5). For instance, whereas competence in youth refers to the perceived mastery of technical skills and the ability to develop strategies during sport participation (14, 15), among masters athletes, competence is more closely related to the ability to maintain health, well-being, and a sense of personal accomplishment (17).

Moreover, the model has been expanded to include dimensions such as contribution and risk behaviors, which reflect developmental processes arising from the strengthening of the original characteristics. In other words, when the 5Cs are fostered, an additional “C” Contribution is likely to emerge, understood as the capacity to contribute to one's community, society, family, and oneself. Conversely, behaviors considered risky or problematic tend to be reduced (12, 18, 19).

In recent years, there has been a growing effort to develop instruments capable of operationalizing the 5Cs model across different contexts, including schools, communities, and sport settings. Examples include the Positive Youth Development measure (13, 20), Vierimaa et al.'s 4Cs proposal, which comprises the Sport Competence Inventory for competence, the Sport Confidence Inventory for confidence, the Coach–Athlete Relationship Questionnaire and Peer Connection for connection, and the Prosocial and Antisocial Behavior in Sport Scale for character and caring (16), as well as the 5Cs Battery for Sport (14).

Except for Vierimaa et al.'s proposal, whose psychometric properties have not been empirically examined (16), the remaining measures have demonstrated satisfactory evidence of validity and reliability, contributing to advances in the operationalization of Positive Youth Development among children, adolescents, and young adults (13, 14). Within the sport context, although only a limited number of instruments are available, they were specifically developed to capture positive developmental experiences during youth, with items designed to reflect manifestations of competence, confidence, connection, caring, and character among children, adolescents, and young adults (14).

However, directly transferring these measures to masters athletes raises important conceptual concerns. From a validity perspective, assessment instruments are expected to adequately represent the construct within the target population (21). Although the theoretical dimensions of competence, confidence, connection, character, and caring may remain relevant across the lifespan, evidence suggests that their behavioral manifestations and subjective meanings vary according to developmental stage and life context (4, 22). For masters athletes, sport participation is intrinsically associated with healthy aging, health maintenance, accumulated life experience, multiple social roles, and long-term engagement in physical activity (23). Consequently, adapting instruments originally designed for youth may fail to capture essential aspects of positive development that are unique to this population.

Although existing measures provide an important theoretical foundation, no instrument has been developed specifically to operationalize Positive Development among masters athletes. Current measures were intentionally designed to represent positive development during youth rather than adulthood, and their items reflect developmental tasks and experiences characteristic of younger populations. Consequently, directly adapting these instruments may fail to capture important aspects of positive development that emerge during adulthood and the aging process.

Furthermore, the 5Cs model and most of its assessment instruments have been developed and validated primarily in WEIRD (Western, Educated, Industrialized, Rich, and Democratic) populations (24, 25). Although Brazil shares certain characteristics with Western societies, it also presents important sociocultural particularities that distinguish it from the populations in which these measures were originally developed. Applying theoretical models and assessment instruments without considering these developmental and cultural specificities may compromise content validity and reduce the fairness and inclusiveness of psychological assessment, particularly for underrepresented populations (26). Therefore, the present study sought to develop an instrument grounded in the meanings that Brazilian masters athletes attribute to the dimensions of positive development.

Some researchers have suggested that development among masters athletes is associated with learning processes grounded in autonomy, the valuing of experience, practical relevance, and self-reflection (7, 27). In the sport context, this implies recognizing masters athletes as active agents in their own learning processes, who seek personal meaning in their participation and value relationships characterized by respect, empathy, and collaboration with coaches and peers (28–30). Additional studies reinforce this perspective, demonstrating that coaches who adopt athlete-centered approaches, based on active listening, flexibility, and co-learning, contribute to athletes’ psychosocial development (30, 31).

Within this context, studies integrating qualitative and quantitative approaches to understand how masters athletes perceive their own psychosocial development, and how these meanings relate to the 5Cs dimensions and their extensions remain scarce. Therefore, the present study has two main objectives: (1) to examine masters athletes’ perceptions of competence, confidence, connection, caring, character, contribution, and risk behaviors through interviews and qualitative analyses; and (2) to develop and estimate content validity evidence for a measure designed to assess these dimensions in the context of masters sport, thereby contributing to the theoretical and methodological advancement of sport psychology and to the consolidation of positive development models across the lifespan.

## Method

### Study 1. Interviews with masters athletes

#### Participants

The sample comprised 25 masters athletes from different sports, including swimming, basketball, cycling, and triathlon. Participants were aged between 30 and 74 years (M = 50.60; SD = 12.16), and self-identified as female (53.85%) and male (42.32%). All participants resided in the states of São Paulo, Rio de Janeiro, and Minas Gerais, Brazil.

### Instrument

#### Semi-structured questionnaire

The interview guide consisted of: (1) questions aimed at characterizing the sample, such as age, gender, sport practiced, and place of residence; and (2) questions designed to understand the athletes’ perceptions of the 5Cs model, including: “How do you define competence in the sports context?”, “What does confidence mean in the sports environment?”, “How do you explain the concept of connection in sport?”, “How would you define care?”, “How would you define character within the sports environment?”, “What does the word ‘contribution’ mean to you?”, “What are the main reasons that lead you to dedicate yourself to and remain engaged in sport?”, and “In your view, what are the main risk behaviors associated with masters athletes?”

The questions were administered in a standardized manner to all participants. When athletes had difficulty providing practical examples of the constructs being discussed, the interviewer used follow-up questions to elicit such responses. For example: “Can you think of a time when you felt confident while practicing your sport? What was that experience like?”

### Procedures

All ethical procedures were conducted in accordance with the guidelines of Resolution No. 466/2012 of the Brazilian National Health Council. Data collection began only after approval from the Research Ethics Committee of São Francisco University, and participation was contingent upon the provision of informed consent.

The first participant was recruited through contact established via LinkedIn. Following this initial interview, a snowball sampling strategy was employed, whereby subsequent participants were referred to and contacted the research team via WhatsApp.

Interviews were conducted via Google Meet and lasted approximately 15 min on average. All sessions were audio-recorded for subsequent transcription and securely stored on Google Drive. Recordings were deleted after the completion of transcription, as previously communicated to participants.

Throughout the sessions, the following materials were used: (1) a sociodemographic questionnaire to collect information on age, gender, sport modality, place of residence, among other variables; and (2) a semi-structured interview guide designed to capture athletes’ perceptions of competence, confidence, connection, caring, character, contribution, and risk behaviors.

### Data analysis

The data analysis was conducted using an exploratory qualitative approach with the aim of identifying semantic patterns and relationships between the meanings expressed in the interviews, using a thematic approach. The process involved multiple steps, combining AI-assisted analysis with manual interpretation based on the 5Cs of Positive Development model.

Initially, all interviews were fully transcribed and imported into Requalify.ai (32), an artificial intelligence, supported qualitative analysis platform used to assist in the organization and preliminary exploration of textual data. The platform was used exclusively as an analytical support tool, while all interpretative decisions remained the responsibility of the research team.

The first step consisted of familiarizing oneself with the dataset through repeated readings of the interview transcripts and the generation of AI-assisted summaries. These summaries were used to identify recurring ideas and facilitate an initial understanding of the corpus. Subsequently, Requalify.ai was used to generate preliminary labels representing concepts, experiences and meanings frequently expressed by the participants. These AI-generated labels were not adopted automatically; rather, they were critically analyzed by the researchers, who examined their relevance, grouped overlapping categories, removed redundancies, and refined their definitions.

After this process, the researchers performed a thematic coding procedure in which the refined categories were organized according to conceptual similarities and interpreted in relation to the theoretical dimensions underlying the study. The coding process involved repeated comparisons between the participants’ narratives, the categories, and the conceptual definitions of the 5Cs model. Discrepancies in interpretation were discussed among the researchers until a consensus was reached on the category definitions and thematic organization.

To further explore the relationships between the identified categories, a similarity network analysis available in Requalify.ai was performed. This procedure allowed the visualization of co-occurrences between themes and provided additional information about the conceptual proximity between the categories. The resulting networks were interpreted as complementary analytical resources, not as isolated evidence. In this way, artificial intelligence served as a tool for organizing and exploring the data, while thematic interpretation remained a process conducted by the researchers.

## Results

**Figure 1:**
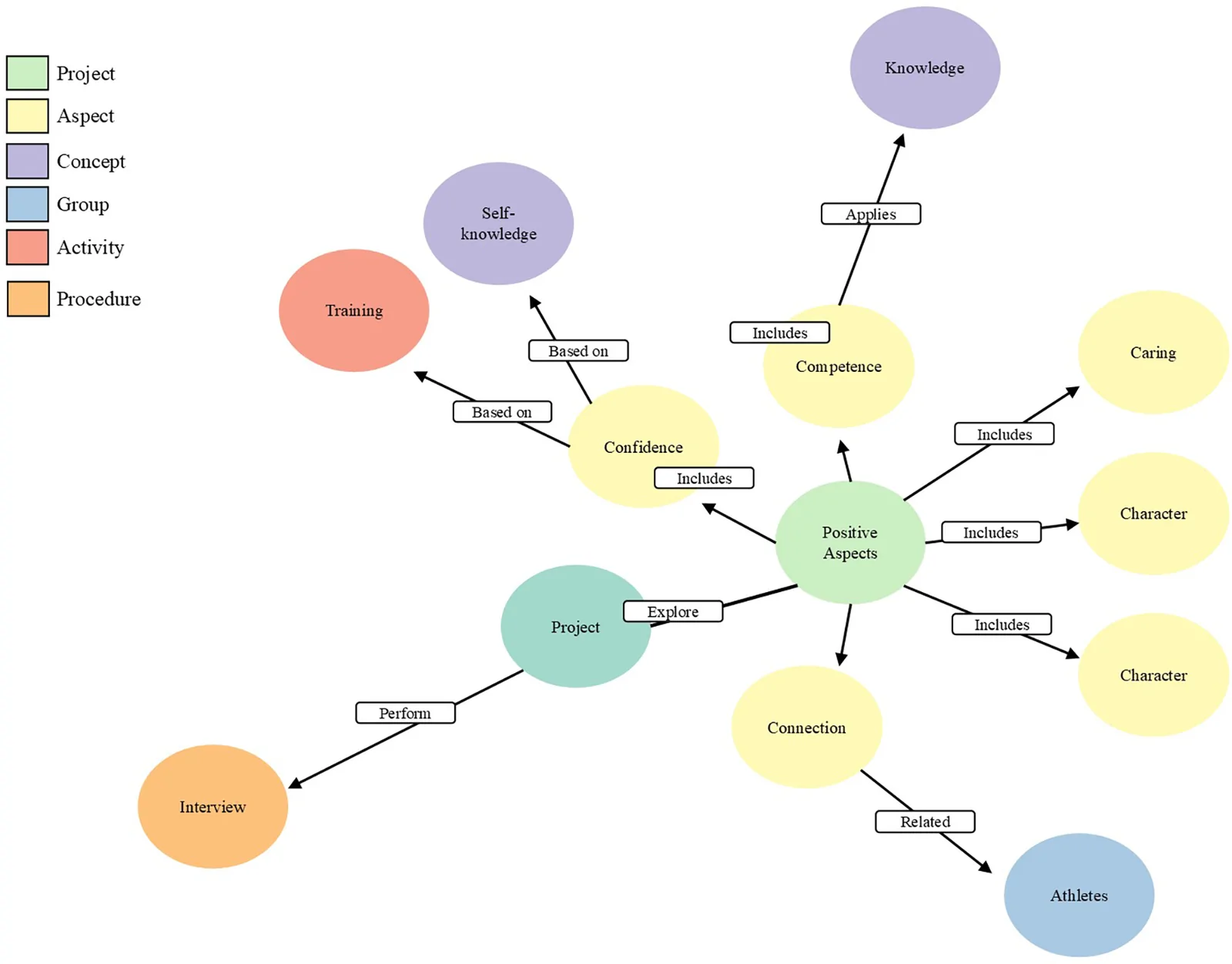
Knowledge network.

The interviews conducted with masters athletes provided a comprehensive overview of their sport participation, highlighting themes such as competence, confidence, connection, caring, character, and contribution. According to the knowledge network generated during the summarization stage, risk behaviors did not emerge as a prominent aspect in the athletes’ accounts (see Figure 1).

Based on Figure 1, the present study aims to explore positive aspects derived from the interviews conducted with masters athletes. These positive aspects include competence, confidence, connection, caring, character, and contribution, the core Cs of the model. Competence was also associated with the application of knowledge. Confidence was described as developed through training and grounded in self-awareness. Connection was reflected in the relationships established among athletes, fostering mutual support, while caring was associated with attention to health and well-being.

These findings are further supported by the results of the category suggestion analysis. In the initial output, 20 categories were identified for the project: human development and values through sport; risk behavior in this age group; contribution and positive role models; risk behaviors in the masters age group; caring as a lifestyle; the importance of self-awareness and personal limits; self-confidence and performance; competence as dedication and discipline; solidarity in sport; contribution and mutual support; motivation and personal fulfillment; caring and sustainability in sport participation; values learned through sport; competence and dedication in sport; connection and interpersonal relationships; character in sport; personal transformation through sport; social connection and interpersonal relationships; caring and prevention in sport participation; and competence and self-awareness.

However, as observed in these descriptions, there were redundancies among categories. Therefore, a subsequent step was conducted by the researchers, who examined the possibility of grouping or excluding certain categories. This process resulted in a refined set of 10 categories, as presented in Table 1.

**Table 1:** Description of categories identified in the interviews.

Category (tag)	Description
Positive development through sport	Athletes perceive sport as a tool for positive development encompassing cognitive, affective, and social domains. Sport participation is viewed as essential for promoting health, well-being, and the development of values such as discipline and resilience.
Competence in sport	Competence refers to the individual ability to apply knowledge acquired through sport participation. Athletes highlight the importance of setting personal goals and engaging in effective training to enhance perceived competence.
Confidence in sport	Confidence is considered essential for sport performance and is defined as the belief in one's ability to achieve desired goals. Its development is associated with training and self-awareness, serving as a key factor in coping with challenges and risks inherent to sport participation.
Connection in sport	Social connection is regarded as fundamental in sport, fostering socialization and mutual support, even in individual sports. Interpersonal relationships vary across competitive contexts, highlighting the importance of meaningful connections that help athletes overcome personal challenges.
Caring in sport	Caring is framed in terms of health and safety, emphasizing the importance of self-care practices, such as regular check-ups and respecting bodily limits, to prevent injuries. Awareness of risks associated with sport participation is particularly important in higher-risk modalities.
Character in sport	Character is described as a set of ethical values guiding athletes’ behavior, including honesty, respect, and sportsmanship. It involves acting ethically, respecting peers, and supporting others, regardless of competitive outcomes.
Contribution	Contribution is understood as helping and inspiring others across family, social, and sport contexts. Athletes emphasize the importance of serving as role models of discipline and health, fostering a more supportive and positive environment among peers.
Risk behavior in masters athletes	Risk behaviors are primarily associated with a lack of bodily awareness, exceeding personal limits, and insufficient professional guidance, which may compromise athletes’ health and safety.
Motivation and personal fulfillment	Key motivations for sport engagement include the pursuit of personal fulfillment and quality of life. Participants report that overcoming challenges and achieving personal goals sustain their motivation and continued involvement in sport.
Personal transformation through sport	Participants suggest that sport participation not only promotes physical health but also contributes to personal development, emphasizing solidarity and the formation of meaningful relationships among athletes.

The findings presented in Table 1 support the notion that the 5Cs model, extended to include contribution and risk behaviors is also applicable within the context of masters sport. Overall, positive development through sport was associated with the promotion of skills and competencies that contribute to a healthy life and higher levels of well-being, as illustrated by Participant 7: “*It is the foundation of life for me. Sport is a tool for human development in all aspects cognitive, socio-emotional, and physical.”* Similarly, Participant 21 stated: “*My main motivation for engaging in sport is the pursuit of health and well-being. Sport participation gives me energy; I feel less stressed, more engaged, and more motivated in other areas of my life.”*

Achieving such positive development entails strengthening the 5Cs, which, in turn, fosters contribution and reduces risk behaviors. Contribution, as described by participants, is associated with prosocial actions such as helping others, sharing, and promoting collective well-being. For instance, Participant 3 reported: “*Contribution is about acting in ways that promote the common good. It improves the environment not only for me individually but, more importantly, helps the group function in a healthier and more appropriate way. In the sport context, it means fostering a more enjoyable environment and ensuring that people have the right to access sport.”* Likewise, Participant 12 described contribution as “*the ability to add value whether within the team, family, or society. It involves helping others and using sport to inspire, guide, and encourage those around you.”*

**Figure 2:**
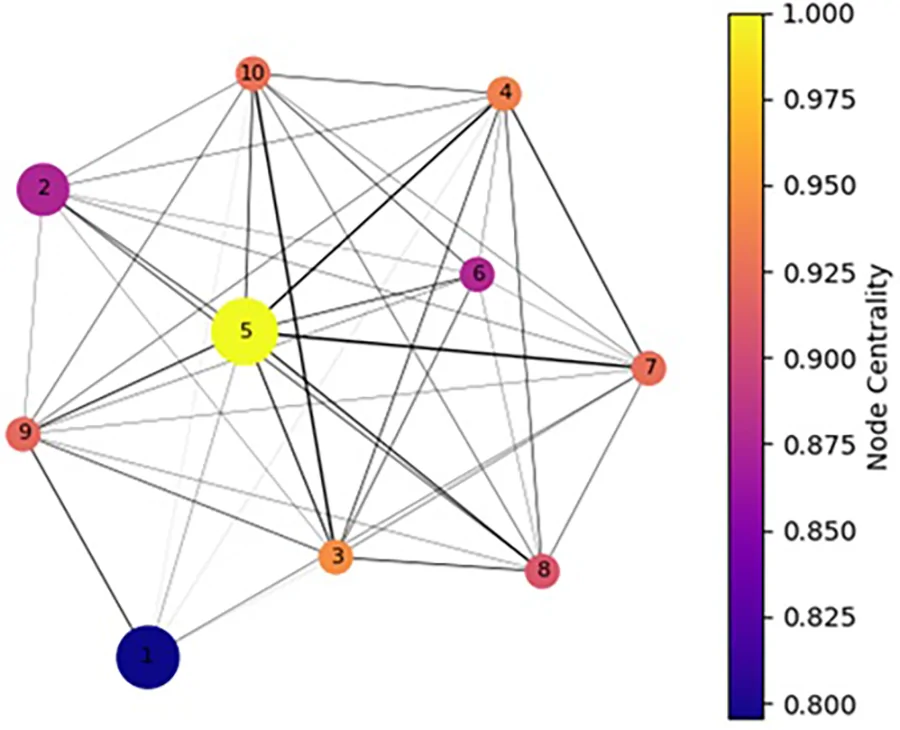
Similarity network. 1 = risk behavior; 2 = contribution; 3 = confidence in sport; 4 = motivation and personal fulfillment; 5 = positive development through sport; 6 = character in sport; 7 = personal transformation through sport; 8 = connection in sport; 9 = caring in sport; 10 = competence in sport.

Finally, a similarity network analysis was conducted to examine how the 10 identified categories relate to one another. As illustrated in Figure 2, positive development emerged as the central node in the network, connected to all identified categories. In other words, the attainment of the 5Cs appears to be associated with positive development, which, in turn, enhances contribution, promotes personal transformation, increases motivation and engagement in sport, and reduces behaviors considered risky. Additionally, the visualization indicates that risk behavior was primarily associated with caring in sport participation and excessive confidence, whereas contribution was connected to all categories except risk behavior.

### Study 2. Item development and content validity evidence

#### Item development

Items designed to assess competence, confidence, connection, caring, character, contribution, and risk behaviors were developed based on the interview findings described in the previous study, as well as on the masters sport literature. Initially, 20 items were generated for each of the 5Cs dimensions. Regarding the outcome-related dimensions (i.e., contribution and risk behaviors), 15 items were developed for each.

It is important to note that the items for the 5Cs were constructed to ensure a balanced representation of positive and negative poles. Specifically, for each positively worded item reflecting a given “C,” a corresponding negatively worded item was also developed. This approach was adopted to capture the full continuum of each construct and to minimize acquiescence bias, that is, the tendency of respondents to systematically agree with statements regardless of their content (33). By incorporating both positively and negatively phrased items, the instrument seeks to encourage more careful response processing and improve the quality of self-reported data (33, 34). This strategy was not applied to the dimensions of contribution and risk behaviors, for which ten positively worded items and five negatively worded items were created, given the conceptual characteristics of these outcomes and the greater emphasis on identifying the presence of adaptive and maladaptive behavioral indicators.

#### Expert review

The evaluation of the items by subject-matter experts was conducted by three judges: two psychologists holding doctoral degrees and specializing in psychological assessment and sports psychology, and a physical education professional with a doctorate specializing in positive development among older adults; they resided in the Brazilian states of São Paulo, Pernambuco, and Paraná. In summary, all judges possessed expertise in sports psychology and psychometrics, with experience in both research and applied settings. Furthermore, they did not participate at any stage in the discussions regarding the 5Cs model or the development of the items.

Item evaluation was conducted using the Content Validity Coefficient (CVC) across three criteria: Clarity of Language (CL), Practical Relevance (PR), and Theoretical Relevance (TR). Judges rated each item against these three criteria using a five-point Likert scale, ranging from 1 (very low) to 5 (very high). To calculate the CVC, responses were converted into values ​​between 0 and 1, following the procedure proposed by Hernández-Nieto. First, the mean of the ratings assigned by the experts to each item was calculated; this value was then divided by the scale's maximum score (5), yielding the content validity index for each criterion. The interpretation of the coefficients followed a more stringent criterion than that proposed by Hernández-Nieto (35), who considers values above 0.80 as acceptable. In the present study, items with values below 0.90 on at least one criterion were excluded.

For the balanced items within the 5Cs, paired items were also excluded when one of the pair did not meet the established criteria. For example, items such as “*I have difficulty believing in my improvement”* and “*I believe I can improve my skills”* were treated as paired opposites, and both could be removed if one failed to meet the threshold.

#### Target population evaluation

The administration of the items to the target population was conducted qualitatively, with the aim of evaluating item adequacy in terms of meaning, difficulty, and comprehensibility, as well as the clarity of the instrument's instructions (36). This stage included 15 masters athletes aged between 29 and 70 years (M = 51.67, SD = 12.22), residing in different states in the Southeast and South regions of Brazil: São Paulo (40%), Rio de Janeiro (33.3%), Paraná (20%), and Minas Gerais (6.7%).

Among the participants, nine self-identified as women and six as men, with the majority reporting having completed graduate-level education (60%). The athletes practiced a range of sports, including athletics, swimming, and judo.

#### Procedures

The study was submitted to and approved by the Research Ethics Committee of Universidade São Francisco. Participants (both experts and athletes) were recruited through social media and the researchers’ professional networks, via an invitation containing a link to the instrument hosted on a Google Forms survey.

The form administered to the experts focused on the evaluation of the Content Validity Coefficient (CVC), whereas the version directed to the target population was designed in a more open format, allowing participants to provide feedback, raise questions, and offer suggestions. Participants were assured of the confidentiality of their information and their right to withdraw at any time, in accordance with the guidelines of Resolution No. 466/2012 of the Brazilian National Health Council. Participation in both stages of the study was contingent upon providing informed consent, and the average time required to complete the forms was approximately 20 min.

## Results

The results of the initial content validity evidence are presented in Table 2, focusing on the items related to the 5Cs. Based on the adopted criteria, the first round of analysis led to the exclusion of three items from each dimension (items 6, 12, and 15 for competence; 4, 6, and 12 for confidence; 4, 9, and 18 for connection; and 3, 8, and 14 for character), with the exception of caring, for which four items (15, 16, 17, and 18) were excluded (CVC < 0.90 on at least one of the evaluated criteria).

**Table 2:** Content validity coefficient of the 5Cs.

Competence	CL	PR	TR	Confidence	CL	PR	TR	Connection	CL	PR	TR
1	1	1	1	1	1	1	1	1	1	1	1
2	1	1	1	2	1	1	1	2	0,93	1	1
3	1	0,93	0,93	3	1	1	1	3	1	1	1
4	0,93	0,93	0,93	4	0,87	1	1	4	0,87	0,93	1
5	0,93	1	1	5	1	1	1	5	1	1	1
6	0,93	1	0,87	6	0,80	1	1	6	1	1	1
7	1	1	0,93	7	0,93	1	1	7	1	1	1
8	1	0,93	0,93	8	0,93	1	1	8	1	1	1
9	1	0,93	0,93	9	0,93	1	1	9	0,87	1	1
10	0,93	1	0,93	10	0,93	1	1	10	1	1	1
11	1	1	0,93	11	0,93	1	1	11	1	1	1
12	0,73	0,93	0,93	12	0,87	1	1	12	1	1	1
13	1	0,93	0,93	13	1	1	1	13	1	1	1
14	1	0,93	0,93	14	1	1	1	14	1	1	1
15	0,93	0,87	0,87	15	1	1	1	15	1	1	1
16	1	0,93	1	16	1	1	1	16	1	1	1
17	1	0,93	0,93	17	0,93	1	1	17	1	1	1
18	1	0,93	0,93	18	0,93	1	1	18	0,67	1	1
19	1	0,93	0,93	19	1	1	1	19	1	1	1
20	1	0,93	0,93	20	1	1	1	20	0,93	1	1

Caring	CL	PR	TR	Character	CL	PR	TR
1	0,93	1	1	1	1	1	1
2	0,93	1	1	2	1	1	1
3	1	1	1	3	0,80	1	1
4	1	1	1	4	1	1	1
5	0,93	1	1	5	0,93	1	1
6	0,93	1	1	6	1	1	1
7	1	1	1	7	1	1	1
8	1	1	1	8	0,80	1	1
9	1	1	1	9	1	1	1
10	1	1	1	10	1	1	1
11	1	1	1	11	1	1	1
12	1	1	1	12	1	1	1
13	1	1	1	13	1	1	1
14	1	1	1	14	0,87	1	1
15	1	1	0,73	15	1	1	1
16	0,93	1	0,73	16	1	1	1
17	0,80	1	1	17	1	1	1
18	0,73	1	0,87	18	0,93	1	1
19	1	1	1	19	1	1	1
20	1	1	1	20	1	1	1

The second round of analysis examined whether the items excluded in the first round had been removed alongside their corresponding paired items. This occurred only within the character dimension. Therefore, for the remaining dimensions, the items that balanced the content of the excluded items were also removed. For example, within the competence dimension, the item “*My energy fluctuates depending on the circumstances of practice”* presented a CVC below 0.90 for theoretical relevance and was therefore excluded from the instrument. Consequently, its paired counterpart, “*I am able to maintain a consistent level of effort during practice,”* was also removed in the second phase, given that the 5Cs instrument was designed to include a balanced set of positively and negatively worded items.

Regarding the evaluation of the contribution and risk behavior items, the coefficients are presented in Table 3. Only one contribution item showed a value below 0.90 for the evaluated criteria; therefore, the final version of this dimension comprised 14 items, while the risk behavior dimension retained all 15 items.

**Table 3:** Content validity coefficient of the contribution and risk behavior.

Contribution	CL	PR	TR	Risk Behavior	CL	PR	TR
1	1	1	1	1	1	1	1
2	0,93	1	1	2	1	1	1
3	1	1	1	3	1	1	1
4	0,93	1	0,93	4	1	1	1
5	1	1	1	5	1	1	1
6	0,93	1	1	6	1	1	1
7	0,93	1	1	7	1	1	1
8	0,93	1	1	8	1	1	1
9	0,80	1	1	9	1	1	1
10	0,93	1	1	10	1	1	1
11	0,93	1	1	11	1	1	1
12	0,93	1	1	12	1	1	1
13	0,93	1	1	13	1	1	1
14	0,93	1	1	14	1	1	1
15	0,93	1	1	15	1	1	1

Regarding the evaluation by the target population, some items received suggestions and were subsequently revised. Specifically, the item “*I apply my knowledge to achieve good results”* was modified to “*I apply my sport-related knowledge to achieve good results.”* The item “*I have difficulty applying my knowledge”* was revised to “*I have difficulty applying my sport-related knowledge in games/competitions.”*

Additionally, the item “*A good diet helps improve my performance”* was adjusted to “*I strive to maintain a healthy diet to improve my sport performance,”* and the item “*Other factors impact my performance more than diet”* was modified to “*Other factors negatively affect my performance more than diet.”*

## Discussion

The findings of this study reinforce that masters sport constitutes a context for positive development in adulthood, in which athletes not only maintain sport participation but also strengthen personal, emotional, cognitive, and relational competencies aligned with the 5Cs model. Qualitative analyses suggest that athletes perceive sport as a space for human growth, continuous learning, and personal fulfillment, which is consistent with previous studies highlighting the psychosocial potential of masters sport (6, 7, 27).

The central category identified as “*positive development through sport”* integrates and supports the remaining dimensions, reflecting the perception that sport participation promotes not only health and well-being but also integrity, empathy, responsibility, self-awareness, and social belonging, in addition to reducing risk behaviors. This perspective expands the understanding of Positive Youth Development (PYD) to the adult context, suggesting that sport continues to function as a developmental environment that supports healthy development and enhances well-being, even beyond adolescence and the transition into adulthood (12, 19).

The results also indicate that the dimensions of the 5Cs assume context-specific meanings in adulthood. For example, caring is associated with health and safety among masters athletes, functioning as a form of awareness regarding the risks inherent in sport participation. This differs from the conceptualization of caring in PYD, where it reflects compassion and empathy toward others and oneself (12, 13, 15).

Similarly, the concept of risk behavior differs across developmental stages. In PYD, it is typically associated with outcomes such as low self-esteem, school dropout, and substance use (12). In contrast, among masters athletes, risk behavior was primarily reflected in the lack of personal limits, excessive physical exertion, engagement in movements that may lead to avoidable injuries, and the absence of adequate professional supervision.

Other Cs demonstrated similarities with those identified in the PYD framework. For example, confidence emerged as being associated with self-awareness and persistence in the face of challenges, suggesting that training and accumulated experience strengthen individuals’ belief in their own capabilities. However, many athletes also reported that excessive confidence may increase the risk of exceeding physical limits, a relationship observed in the similarity network linking confidence, caring, and risk behavior.

Competence, in turn, was associated with both technical mastery and a sense of personal accomplishment, particularly in terms of applying knowledge during sport participation. At the same time, the establishment of goals was described as essential for adapting training routines and enhancing perceived competence. These findings partially support PYD perspectives (12, 13) and are consistent with qualitative research on competence in masters sport (17).

Study 2, focused on item development and the analysis of content validity evidence, indicated that most items demonstrated adequate quality, as reflected in the consistency of expert evaluations based on the Content Validity Coefficient and the qualitative review conducted with the target population. These findings suggest that the content derived from the interviews was appropriately translated into observable items, ensuring semantic coherence between athletes’ reported experiences and their psychometric operationalization within the proposed instrument. Overall, the results support the retention of the majority of items, despite the exclusion of some, providing initial evidence of content-based validity for the 5Cs measure, as well as for the contribution and risk behavior scales, in accordance with the standards established by the American Educational Research Association (AERA), American Psychological Association (APA), and National Council on Measurement in Education (NCME) (37).

Thus, the integration of the two studies enabled theoretical and methodological advancements in the field of masters sport psychology. Theoretically, this work expands the scope of the 5Cs model by demonstrating its applicability to masters athletes, highlighting that sport continues to promote emotional, physical, social, and cognitive competencies beyond adolescence and young adulthood (12–24 years). Methodologically, the study contributes by developing an initial measurement instrument grounded in the lived experiences of masters athletes, thereby supporting future empirical research on positive development across the lifespan.

In summary, the findings indicate that masters sport constitutes a context that fosters positive development, in which the dimensions of the 5Cs model remain active and interdependent, assuming meanings that reflect individuals’ relationships with sport participation, peers, and their health and well-being. Despite these contributions, some limitations should be acknowledged. The relatively small sample size and the predominance of athletes from the Southeast region of Brazil may limit the generalizability of the findings. Furthermore, participants were recruited using a snowball sampling strategy, which may have increased the likelihood of individuals from similar social networks and sporting contexts. In other words, the strategy adopted may have decreased the diversity of perspectives represented in the qualitative data.

Another limitation concerns the reliance on self-reported perceptions of positive development. The theoretical model underlying the instrument is composed of constructs considered socially valued, which may result in responses that reflect socially accepted views rather than real experiences. Although methods to minimize the social desirability of specific items were not applied, techniques were employed to minimize acquiescence, which refers to the behavior of agreeing with items regardless of their content. To this end, the strategy of constructing items with positive and negative poles for each construct was adopted. However, for the present study, no analyses were performed to support the instrument's ability to control response bias, considering the low number of participants.

This limitation is directly related to the main objective of the study, which was to develop a measurement proposal and provide initial evidence based on content validity procedures. Although content validity represents a fundamental step in the development of instruments, it does not provide sufficient evidence about the internal structure, reliability, measurement invariance, or relationships with external variables. Therefore, the results should be interpreted as preliminary support for the representativeness and relevance of the proposed items, and not as evidence of the overall psychometric quality of the measure.

Furthermore, although the use of artificial intelligence tools, such as Requalify.ai, has facilitated the organization and analysis of large volumes of textual data, interpretations and analytical decisions have remained dependent on the judgment of researchers. Additionally, AI-based tools operate using algorithmic models that can influence the processes of categorization, synthesis, and grouping of qualitative information. Therefore, transparency in the procedures adopted and continuous human supervision are fundamental aspects to ensure methodological rigor and the proper interpretation of results.

Future research should seek to expand the sample size and look for evidence of validity for the measure. That is, to test the factorial structure, explore the ability to control for acquiescent response bias, verify the relationship with other variables, and examine how different characteristics of athletes (e.g., gender, sport, and years of experience) are associated with positive development.

## Data Availability

The raw data supporting the conclusions of this article will be made available by the authors, without undue reservation.
